# An Incomplete Superficial Palmar Arterial Arch: Clinical Considerations of the Modified Allen's Test

**DOI:** 10.7759/cureus.82356

**Published:** 2025-04-16

**Authors:** Takutoshi Inoue, Toru Yamamoto, Joe Iwanaga

**Affiliations:** 1 Department of Anatomy, Teikyo University School of Medicine, Tokyo, JPN; 2 Division of Dental Anesthesiology, Graduate School of Medicine and Dental Sciences, Niigata University, Niigata, JPN; 3 Department of Neurosurgery, Tulane Center for Clinical Neurosciences, Tulane University School of Medicine, New Orleans, USA

**Keywords:** anatomy training body, cadaver, circulation management, hemodynamic monitoring, modified allen's test, superficial palmar arch

## Abstract

The modified Allen's test (MAT) is a non-invasive test for evaluating the circulation of the hand, including the superficial palmar arch (SPA). However, although there are many variations of SPA, it cannot be said that it is widely recognized in clinical practice. Herein, we report an incomplete SPA observed in the right hand of an 83-year-old male cadaver. Notably, the superficial palmar branch of the radial artery ran superficial to the abductor pollicis brevis muscle, and no median artery was identified. Six common palmar digital arteries, that is, four from the ulnar artery and two from the radial artery, were observed. This anatomical configuration lacked an anastomotic connection between the radial and ulnar arteries, suggesting insufficient collateral circulation. As a result, performing the MAT in such a case may yield a "positive" result, indicating inadequate collateral blood flow. However, in the context of an incomplete SPA, this result may reflect a true anatomical limitation rather than a pathological arterial obstruction. Therefore, clinicians must consider underlying anatomical variations when interpreting MAT results. Supplemental diagnostic tools, such as Doppler ultrasonography or pulse oximetry, may help distinguish between anatomical variants and vascular pathology. Recognizing these variations is essential for accurate circulatory assessment and safe hemodynamic monitoring during anesthesia and other clinical interventions.

## Introduction

The modified Allen's test (MAT) is a non-invasive method for evaluating the hand's circulation [1]. This test is commonly used to confirm the presence of compensatory circulation in the palm of the hand before puncturing the radial artery for hemodynamic monitoring during the administration of general anesthesia and in the intensive care unit [2]. A positive MAT result indicates inadequate collateral circulation. The radial and ulnar arteries are distributed in the forearm and hand, and these arteries anastomose in the hand to form a palmar arch, called the superficial palmar arch (SPA), also known as the superficial palmar arterial arch [1]. However, there are many variations of the SPA, which can be classified as complete or incomplete depending on the presence or absence of anastomosis of the two arteries [3,4].

An incomplete SPA is crucial in arterial blood sampling, hand reconstruction surgery, arterial grafts in cardiac bypass surgery, and various microvascular surgeries [4]. In cases of incomplete SPA, collateral blood flow to the hand may be insufficient if one artery is occluded or compromised. This anatomical variation may render the MAT less reliable, as the test assumes the presence of a complete anastomosis for accurate assessment. Most reports on incomplete SPA [3,4] described surgical procedures, but only a few discussed perioperative management. In this article, we present a case of incomplete SPA from the perspective of circulatory management during general anesthesia.

## Case presentation

This case study was conducted in accordance with the guidelines stipulated in the Declaration of Helsinki. We followed the guidelines for research involving cadavers established by the Japanese Association of Anatomists.

**Figure 1:**
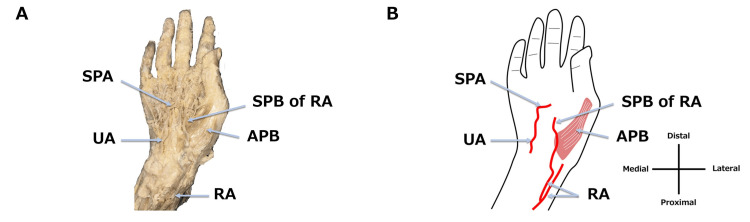
Incomplete SPA (right hand). A photograph (A) and schematic diagram (B) of an incomplete SPA on the right hand. The incomplete SPA is formed by the UA and the SPB of RA which branches from the RA and runs superficial to the APB. SPA: superficial palmar arch; UA: ulnar artery; SPB of RA: superficial palmar branch of the radial artery; RA: radial artery; APB: abductor pollicis brevis Image Credits: Takutoshi Inoue

**Figure 2:**
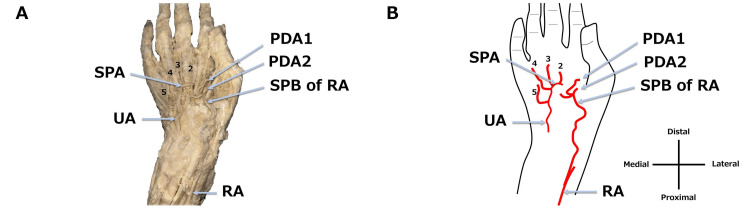
Incomplete SPA after the removal of the thenar muscles (right hand). A photograph (A) and schematic diagram (B) show the removal of the thenar muscles including the APB. SPA: superficial palmar arch; UA: ulnar artery; RA: radial artery; 2-5: common palmar digital arteries; PDA1, PDA2: palmar digital arteries to the first digit; APB: abductor pollicis brevis Image Credits: Takutoshi Inoue

We found an incomplete right SPA during an anatomy training session at Teikyo University School of Medicine in 2024. This case involved an 83-year-old man with a height of 160 cm, a weight of 48 kg, and a body mass index of 18.8 kg/m^2^. The cause of death was senility. Before the anatomical dissection, formalin was injected into the left femoral artery, and the cadaver was then preserved with alcohol and formalin. During dissection, the right upper limb was amputated, and the skin of the palm was removed. The palmar aponeurosis was then removed, and the SPA was dissected. The right SPA was incomplete, with no connection between the superficial palmar branch of the radial artery (SPB of RA) and the terminal branch of the ulnar artery (Figure 1). The SPB of RA ran superficial to the abductor pollicis brevis (APB). However, when the thenar muscles, including the APB, were removed, the median artery was absent (Figure 2). Furthermore, four palmar digital arteries from the ulnar artery and two palmar digital arteries arising from the SPB of RA were identified (Figure 2).

The classification of incomplete SPA has been reported by researchers. This case can be evaluated using the Coleman and Anson classification and the Gnanasekaran and Veeramani classification because radial arteries, ulnar arteries, and common palmar digital arteries were observed. According to the classification of incomplete SPAs reported by Coleman and Anson [5,6], our case of incomplete SPA without a median artery is classified as Type A (radial and ulnar arteries) and is similar to Type 7 (palmar arch, radio-ulnar) of Gnanasekaran and Veeramani's classification [7].

## Discussion

In this report, we described a case of an incomplete SPA. The prevalence of incomplete SPA ranges from 3.6% to 78.5% [5,8]. Our literature review included cadavers and subjects with imaging, and prevalence rates were within this range. The discrepancy in the reported prevalence may be due to different ethnicities examined, sex, sample size, and interpretation of classification [9].

The SPB of RA, one of the contributions of the SPA, usually travels through the thenar muscle [10]. However, as in the present case, patients with recurrent pain in the thenar muscles due to an incomplete SPA, where the SPB of RA passes superficially to the thenar muscles, have been reported. This condition may be detected incidentally in medical practice [10]. Though not a well-established syndrome, its presence may be clinically relevant in compression neuropathies or surgical approaches to the thenar region. Such anatomical abnormalities may also be discovered accidentally in clinical practice.

**Figure 3:**
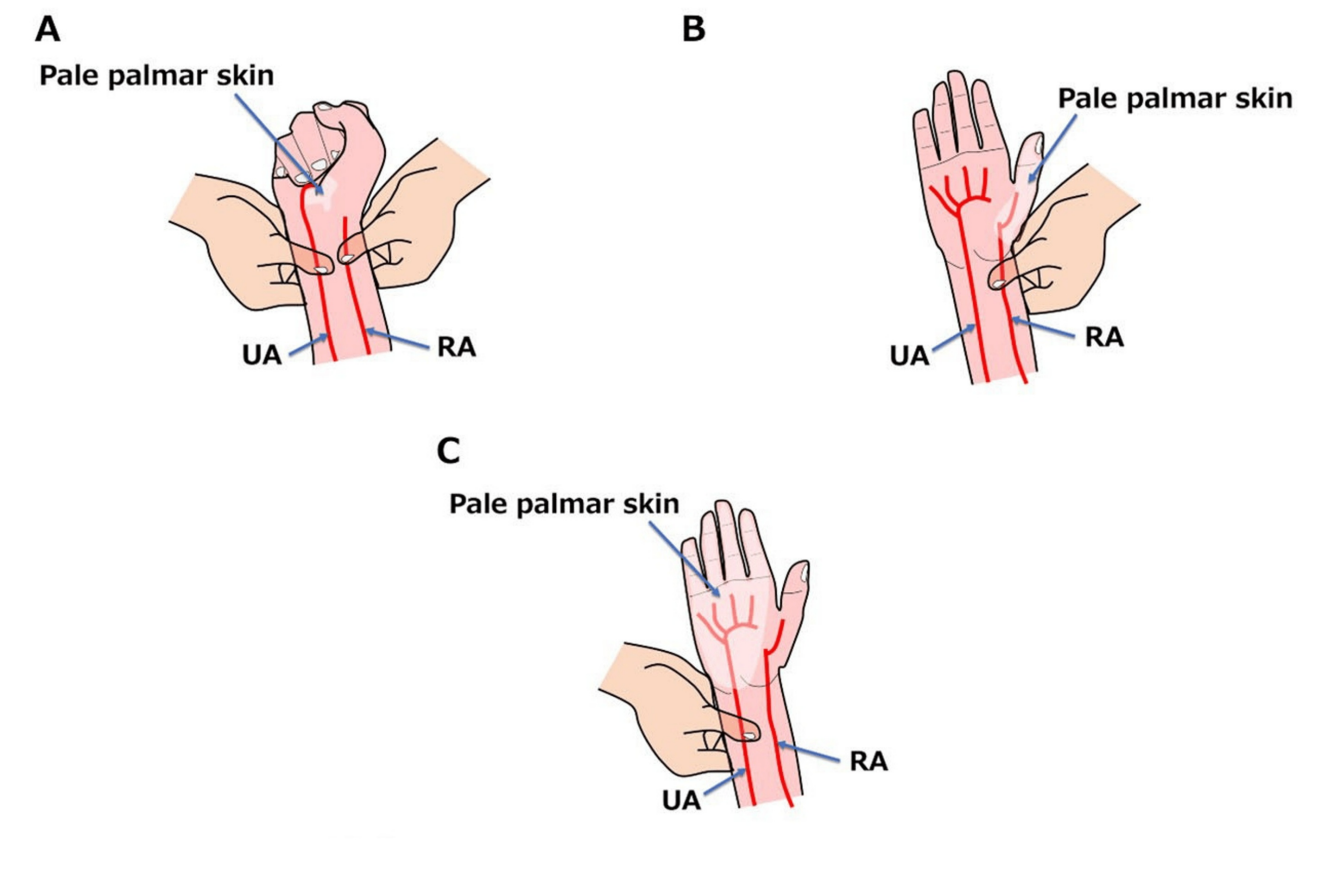
Modified Allen's test in a patient with this variation. (A) Compress both the RA and UA in the patient's wrist and ask the patient to repeatedly clench his or her fist until the skin on the palmar side blanches. (B) While maintaining compression of the RA, blood flow is released through the UA. (C) While maintaining compression of the UA, blood flow in the RA is released. RA: radial artery; UA: ulnar artery Image Credits: Takutoshi Inoue

During the MAT procedure, both the radial and ulnar arteries at the patient's wrist are compressed first to block blood flow; then, the patient is asked to make a fist several times until the skin on the palmar side turns white (Figure 3A). Subsequently, the patient is asked to open their hand, and the pressure on the ulnar artery is released while maintaining compression of the radial artery. If the MAT is performed on a patient with the present variation, blood return to the palmar skin of the first digit may be slow, resulting in a positive test. This could be incorrectly interpreted as an obstruction or inadequacy of blood flow through the ulnar artery (Figure 3B). Therefore, an additional examination involving the compression of the ulnar artery and the release of the radial artery should be conducted. This helps raise a suspicion not only for arterial occlusion but also for the presence of an incomplete SPA (Figure 3C).

Currently, MAT is widely used in the evaluation of the overall condition of patients under anesthesia. However, the frequency of abnormal MAT results ranges from 1% to 27%, and whether it is an effective screening test remains controversial [11]. This is a subjective assessment, subject to interobserver variability and variations in arterial anatomy, which can lead to false-positive or false-negative results [1,11]. To clarify the clinical evidence of positive MAT results, objective tests, including pulse oximetry, plethysmography, Doppler ultrasonography, laser Doppler, and angiography, are suggested [1].

During general anesthesia, it is crucial to constantly monitor the physiological functions of the patient. A pulse oximeter is attached to monitor the respiration. It can also measure peripheral arterial oxygen saturation and pulse rate non-invasively and continuously and can generate a pulse wave as a waveform. The use of a pulse oximeter along with MAT may be useful for detecting incomplete SPA [12]. If a pulse oximeter probe is attached to the first digit and MAT is performed in a patient with this variation, the return of the waveform could be insufficient when the ulnar artery is released while maintaining pressure on the radial artery. Considering that vital sign monitors are generally available outside the operating room, such as in outpatient hospitals, in clinics, and even in general dental practice (in case of sedation and emergency situations), this method can be easily performed anywhere [13]. It has been reported that all patients with indeterminate MAT results had normal pulse oximetry, but it should be noted that the demonstration of normal saturation of pulse oximetry may not ensure adequate tissue perfusion [11].

Recently, non-invasive continuous blood pressure monitoring has enabled the assessment of hemodynamics without the need for performing arterial puncture. Particularly, the ClearSight system (Edwards Lifesciences, Irvine, California, United States) is a monitoring device that allows non-invasive and continuous measurement of arterial blood pressure using the finger cuff method [14]. Furthermore, it can help obtain valuable data, including cardiac output, stroke volume, stroke volume variation, systemic vascular resistance, and systemic vascular resistance index [14]. Moreover, it allows the hemodynamic monitoring of patients with cardiovascular disease and of cases where blood pressure monitoring at the arm or arterial puncture is difficult or blood gas analysis is not required. Easy access to continuous blood pressure monitoring makes having knowledge of the SPA variations even more important. In patients with incomplete SPA, altered perfusion dynamics in the fingers may affect the accuracy or stability of these readings. Though not contraindicated, clinicians should be aware that abnormal waveform morphology or unstable signals may prompt the consideration of an underlying vascular anomaly. Further studies are needed to validate the reliability of these devices in the presence of SPA variants.

In summary, anatomical variations in the SPA may significantly affect the interpretation of bedside tests, such as the MAT, and the performance of non-invasive hemodynamic monitoring devices. When clinicians encounter ambiguous or inconsistent MAT results or delayed waveform return limited to specific digits, the possibility of an incomplete SPA should be considered. In such cases, reliance on the MAT alone may be insufficient, and supplemental objective assessments, such as Doppler ultrasonography, can provide clearer insights. Furthermore, careful consideration should be given to selecting vascular access sites to avoid ischemic complications. Awareness of these anatomical variations and their clinical implications is essential for ensuring accurate evaluation and safe patient management, particularly in perioperative, outpatient, and emergency settings.

## Conclusions

Recognizing the anatomical variations of SPA is crucial in perioperative management. The present case report of incomplete SPA highlights the importance of performing an objective test in addition to the traditional MAT. An incomplete SPA, such as the one observed in this cadaveric study, may lead to a false-positive MAT result, potentially resulting in inappropriate clinical decisions such as unnecessary avoidance of radial artery cannulation or incorrect selection of an arterial access site. Given the variable prevalence of incomplete SPAs and the inherent subjectivity and limitations of MAT, incorporating additional diagnostic modalities, such as pulse oximetry or Doppler ultrasonography, can enhance the accuracy of circulatory evaluation. For example, if MAT yields an unexpected positive result, follow-up with Doppler ultrasonography may help distinguish true arterial insufficiency from an anatomical variation.

Ultimately, MAT should not be solely relied upon for vascular assessment in patients undergoing procedures that involve the radial artery. Its results must be interpreted within the broader context of anatomical variability. A thorough understanding of hand vascular anatomy is fundamental to ensuring safe and effective cardiovascular monitoring, particularly in anesthesiology, surgery, emergency medicine, and intensive care settings.
